# Supplementary material for: Health and Care Dependency of Older Adults in Dresden, Germany: Results from the LAB60+ Study
Source: Int J Environ Res Public Health. 2022 Sep 18;19(18):11777. doi: 10.3390/ijerph191811777 (PMC9517330; doi:10.3390/ijerph191811777)

Table S1. List of comorbidities given in the LAB60+ questionnaire

Do you have or have you had any of the following conditions? (multiple answers possible)

None	Diabetes
High blood pressure	Heart failure
Coronary heart disease	Hear attack
Osteoarthritis	Chronic pain (e.g. back pain)
Osteoporosis	Rheumatism
Depression	Stroke
Lung disease (e.g. COPD)	Dizziness
Dementia (e.g. Alzheimer's)	Cancer (in the past 5 years)
Eye disease (e.g. cataract)	Hearing impairment
Incontinence	Others, namely:

Evaluation of degree of care in Germany
(from Bundesministerium für Gesundheit. Online-Ratgeber Pflege: Pflegegrade. Availabe online: https://www.bundesgesundheitsministerium.de/pflegegrade.html (accessed on 12.10.2021).

To determine how independent a person in need of care is, the assessor takes a close look at the following six areas of life:

Module 1 "Mobility": the assessor looks at physical mobility. For example, can the person get up and walk from the bed to the bathroom on his or her own? Can he or she move around independently within his or her own four walls, is climbing stairs possible?

Module 2 "Mental and communicative abilities": This area includes understanding and talking. For example: Can the person concerned orient himself in time and space? Does he or she understand facts, recognize risks and can he or she hold conversations with other people?

Module 3 "Behavioral and psychological problems": This includes restlessness at night or fears and aggression, which are stressful for the person in need of care, but also for their relatives. This is also taken into account if there are defensive reactions to nursing measures.

Module 4 "Self-care": For example, can the applicant wash and dress him/herself, can he/she go to the toilet independently and eat and drink?

Module 5 "Dealing independently with requirements and stresses caused by illness or therapy - and coping with them": The assessor looks at whether the person concerned can, for example, take medication him/herself, measure blood sugar independently, use aids such as prostheses or a walker, and see a doctor.

Module 6 "Shaping everyday life and social contacts": For example, can the person concerned organize his or her daily routine independently? Can he or she come into direct contact with other people or go to the skat club without help?

For each criterion in the above-mentioned areas of life, the assessors determine the degree of independence of the person in need of care, usually on the basis of a point value between 0 (person can perform activity without a helping person, if necessary with aids) and - 3 (person cannot perform the activity, not even in parts). At the end, the points with different weighting are combined to a total value, which stands for one of the five degrees of care. The five care grades are graduated: from minor impairments of independence or abilities (care grade 1) to the most severe impairments of independence or abilities that are accompanied by special requirements for nursing care (care grade 5), as shown in Table A2:

Table S2. Description of degree of care

Degree of care	Number of total points	Definition
1	12.5 to <27	minor impairments of independence or abilities
2	27 to <47.5	significant impairment of independence or abilities
3	47.5 to <70	severe impairment of independence or abilities
4	70 to <90	most severe impairment of independence or abilities
5	90 to 100	most severe impairments of independence or abilities with special requirements for nursing care

Table S3. Mean scores of the SF-8 sub- and total scales by 5-year age groups and gender.

	**GH**		**PF**		**RP**		**BP**		**VT**		**SF**		**MH**		**RE**		**PCS-8**		**MCS-8**	
	M	***95%-CI***	***M***	***95%-CI***	***M***	***95%-CI***	***M***	***95%-CI***	***M***	***95%-CI***	***M***	***95%-CI***	***M***	***95%-CI***	***M***	***95%-CI***	***M***	***95%-CI***	***M***	***95%-CI***
60-64 years																				
Women	46.7	(45.9-47.5)	47.4	(46.5-48.3)	47.7	(46.7-48.6	50.9	(49.8-52.1)	49.6	(48.7-50.5)	47.6	(46.4-48.8)	47.2	(45.9-48.5)	46.6	(45.6-47.6)	48.8	(47.7-45.0)	47.5	(46.1-48.9)
Men	46.1	(45.3-47.1)	47.0	(46.0-47.9)	47.3	(46.3-48.3)	50.0	(48.8-51.1)	49.5	(48.6-50.4)	47.0	(45.7-48.3)	48.7	(47.5-49.9)	46.1	(45.1-47.2)	47.6	(46.5-48.7)	48.5	(47.2-49.8)
Total	46.4	(45.8-47.0)	47.2	(46.6-47.9)	47.5	(46.8-48.2)	50.4	(49.6-51.3)	49.5	(48.9-50.2)	47.3	(46.4-48.2)	48.0	(47.1-48.8)	46.4	(45.7-47.1)	48.2	(47.5-49.0)	48.0	(47.1-49.0)
65-69 years																				
Women	45.9	(45.1-46.7)	46.6	(45.6-47.6)	47.6	(46.6-48.6)	50.2	(49.0-51.4)	49.1	(48.1-50.0)	47.4	(46.2-48.5)	47.9	(46.7-49.0)	46.7	(45.8-47.6)	47.9	(46.7-49.1)	48.1	(46.8-49.3)
Men	46.9	(46.1-47.8)	47.3	(46.2-48.3)	47.7	(46.6-48.7)	52.6	(51.4-53.7)	50.3	(49.4-51.2)	49.4	(48.2-50.5)	50.4	(49.3-51.5)	48.2	(47.3-49.1)	48.6	(47.4-49.8)	51.1	(49.8-52.3)
Total	46.4	(45.8-47.0)	46.9	(46.2-47.6)	47.6	(46.9-48.3)	51.3	(50.5-52.1)	49.7	(49.0-50.3)	48.3	(47.5-49.1)	49.1	(48.3-49.9)	47.4	(46.8-48.1)	48.2	(47.4-49.0)	49.5	(48.6-50.4)
70-74 years																				
Women	44.8	(44.0-45.6)	44.2	(43.0-45.4)	45.3	(44.1-46.4)	47.2	(45.8-48.5)	47.5	(46.5-48.5)	45.3	(43.8-46.7)	47.4	(46.1-48.7)	45.8	(44.9-46.8)	44.7	(43.3-46.0)	47.4	(46.0-48.8)
Men	45.1	(44.1-46.1)	45.2	(43.8-46.7)	46.4	(45.1-47.8)	50.2	(48.8-51.6)	49.1	(48.0-50.2)	47.9	(46.4-49.4)	50.9	(49.7-52.1)	47.3	(46.2-48.5)	45.9	(44.3-47.5)	51.1	(49.7-52.4)
Total	44.9	(44.3-45.5)	44.6	(43.7-45.5)	45.8	(44.9-46.7)	48.4	(47.4-49.4)	48.2	(47.5-49.0)	46.4	(45.4-47.5)	48.9	(48.0-49.8)	46.5	(45.7-47.2)	45.2	(44.2-46.2)	49.0	(48.0-45.0)
75-79 years																				
Women	43.3	(42.6-44.1)	41.7	(40.5-42.8)	42.9	(41.7-44.1)	46.2	(44.9-47.4)	46.2	(45.3-47.1)	44.5	(43.3-45.8)	46.4	(45.2-47.6)	44.6	(43.6-45.5)	42.2	(40.9-43.6)	46.6	(45.3-47.9)
Men	44.2	(43.5-45.0)	43.9	(42.8-45.0)	44.8	(43.7-46.0)	48.4	(47.1-49.6)	47.9	(47.1-48.8)	46.7	(45.3-48.0)	49.4	(48.2-50.6)	46.5	(45.5-47.5)	44.2	(42.9-45.5)	49.6	(48.3-51.0)
Total	43.8	(43.2-44.3)	42.7	(41.9-43.5)	43.8	(43.0-44.7)	47.2	(46.3-48.1)	47.0	(46.4-47.6)	45.5	(44.6-46.4)	47.8	(47.0-48.6)	45.5	(44.7-46.2)	43.1	(42.2-44.1)	48.0	(47.1-49.0)
80-84 years																				
Women	41.3	(40.4-42.3)	39.0	(37.7-40.4)	39.6	(38.2-41.0)	43.6	(42.1-45.0)	43.9	(42.9-45.0)	42.7	(41.2-44.3)	45.2	(43.7-46.7)	42.7	(41.4-44.1)	38.5	(37.0-40.0)	45.1	(43.5-46.8)

	GH		PF		RP		BP		VT		SF		MH		RE		PCS-8		MCS-8	
	M	***95%-CI***	***M***	***95%-CI***	***M***	***95%-CI***	***M***	***95%-CI***	***M***	***95%-CI***	***M***	***95%-CI***	***M***	***95%-CI***	***M***	***95%-CI***	***M***	***95%-CI***	***M***	***95%-CI***
Men	43.4	(42.6-44.2)	41.4	(40.1-42.7)	42.3	(41.0-43.6)	47.8	(46.4-49.2)	46.2	(45.2-47.2)	45.5	(44.0-47.0)	48.7	(47.4-50.0)	45.5	(44.3-46.6)	41.8	(40.3-43.2)	48.9	(47.5-50.4)
Total	42.4	(41.7-43.0)	40.2	(39.2-41.1)	40.9	(40.0-42.0)	45.6	(44.6-46.7)	45.0	(44.3-45.8)	44.1	(43.0-45.2)	46.9	(45.9-47.9)	44.1	(43.2-45.0)	40.1	(39.1-41.2)	47.0	(45.9-48.1)
85+ years																				
Women	40.2	(38.9-41.4)	35.7	(33.9-37.5)	35.9	(34.0-37.8)	41.7	(39.7-43.7)	41.6	(39.9-43.3)	40.8	(38.5-43.0)	45.1	(43.1-47.1)	40.6	(38.7-42.6)	34.5	(32.4-36.7)	44.5	(42.3-46.7)
Men	42.1	(41.0-43.1)	38.0	(36.3-39.7)	39.9	(38.3-41.6)	45.6	(43.7-47.4	44.9	(43.6-46.2)	44.2	(42.2-46.2)	48.2	(46.5-49.9)	44.3	(42.6-46.0)	38.5	(36.7-40.3)	48.5	(46.6-50.5
Total	41.2	(40.3-42.0)	36.9	(35.7-38.1)	38.1	(36.8-39.3)	43.8	(42.5-45.2)	43.4	(42.3-44.5)	42.6	(41.1-44.1)	46.7	(45.3-48.0)	42.5	(41.2-43.8)	36.7	(35.3-38.1)	46.6	(45.1-48.1)
Total																				
Women	44.2	(43.8-44.6)	43.3	(42.8-43.9)	44.1	(43.6-44.6)	47.3	(46.8-47.9)	46.9	(46.5-47.4)	45.2	(44.7-45.8)	46.7	(46.2-47.3)	45.0	(44.5-45.5)	43.9	(43.3-44.5)	46.8	(46.2-47.4)
Men	44.9	(44.6-45.3)	44.4	(43.9-44.9)	45.2	(44.7-45.8)	49.4	(48.9-50.0)	48.3	(47.9-48.7)	47.0	(46.4-47.6)	49.4	(48.9-49.9)	46.5	(46.0-46.9)	45.1	(44.5-45.6)	49.6	(49.1-50.2)
Total	44.5	(44.3-44.8)	43.8	(43.5-44.2)	44.7	(44.3-45.0)	48.3	(47.9-48.7)	47.6	(47.3-47.9)	46.1	(45.7-46.5)	48.0	(47.6-48.4)	45.7	(45.4-46.0)	44.5	(44.0-44.9)	48.2	(47.8-48.6)

Note. ***GH*** *General Health* ***PF*** *Physical Functioning* ***RP*** *Role Physical* ***BP*** *Bodily Pain* ***VT*** *Vitality* ***SF*** *Social Functioning* ***RE*** *Role Emotional* ***MH*** *Mental Health* ***PCS*** *Physical Summary Scales* ***MCS*** *Mental Summary Scales*

Table S4. Mean scores of the SF-8 subscales for the LAB60+ population and the German normal sample by age and gender

	GH	PF	RP	BP	VT	SF	MH	RE	PCS-8	MCS-8
**60-69 years**										
Women										
LAB60+	46.4	47.1	47.6	50.6	49.4	47.3	47.4	46.6	48.5	47.6
Normal sample*	45.5	47.4	47.9	48.6	49.4	51.6	50.5	49.0	47.2	51.7
Men										
LAB60+	46.5	47.3	47.6	51.3	50.0	48.0	49.4	47.1	48.3	49.5
Normal sample*	46.0	48.0	48.7	50.9	50.1	52.6	52.5	49.4	48.2	53.4
**70-79 years**										
Women										
LAB60+	44.3	43.4	44.5	47.1	47.0	45.2	47.2	45.4	43.9	47.3
Normal sample*	44.1	45.5	46.0	47.3	48.2	51.6	50.5	48.1	44.8	51.6
Men										
LAB60 +	44.8	44.4	45.6	49.1	48.6	47.5	50.4	47.2	44.9	50.8
Normal sample*	45.0	46.9	48.2	50.0	50.2	52.4	52.9	49.1	47.2	53.7
**80+ years**										
Women										
LAB60+	41.2	38.4	39.1	43.4	43.7	42.6	45.2	42.3	37.9	45.1
Normal sample*	42.5	43.2	43.9	44.7	47.3	51.1	49.7	46.5	41.7	52.3
Men										
LAB60+	43.2	41.0	42.1	47.4	46.0	45.3	48.5	45.1	41.4	48.7
Normal sample*	44.2	46.1	45.5	49.3	44.5	52.4	52.1	48.2	44.5	52.3

**Normal sample of the German population from Ellert et al. 2005*

Table S5. WHO 5 mean weighted scores by 5-year age groups and gender.

	WHO-5	
	Mean	***95%-CI***
60-64 yrs		
Women	58.2	(55.3 - 61.1)
Men	59.2	(56.4 - 62.1)
Total	58.6	(56.6 - 60.7)
65-69 yrs		
Women	60.6	(57.7 - 63.6)
Men	64.9	(62.1 - 67.7)
Total	62.7	(60.7 - 64.7)
70-74 yrs		
Women	57.9	(54.7 - 61.1)
Men	65.9	(62.4 - 69.4)
Total	61.4	(59.0 - 63.8)
75-79 yrs		
Women	54.2	(51.2 - 57.1)
Men	61.9	(58.9 - 64.9)
Total	57.8	(55.6 - 59.9)
80-84 yrs		
Women	48.9	(45.2 - 52.7)
Men	58.3	(54.9 - 61.7)
Total	53.6	(51.0 - 56.2)
85+ yrs		
Women	45.3	(40.4 - 50.2)
Men	57.0	(53.1 - 60.9)
Total	51.8	(48.6 - 54.9)
Total		
Women	55.3	(54.0 - 56.7)
Men	61.4	(60.1 - 62.7)
Total	58.3	(57.3 - 59.2)

Figure S1. Number of household members (including the respondent) in the study population by age and gender

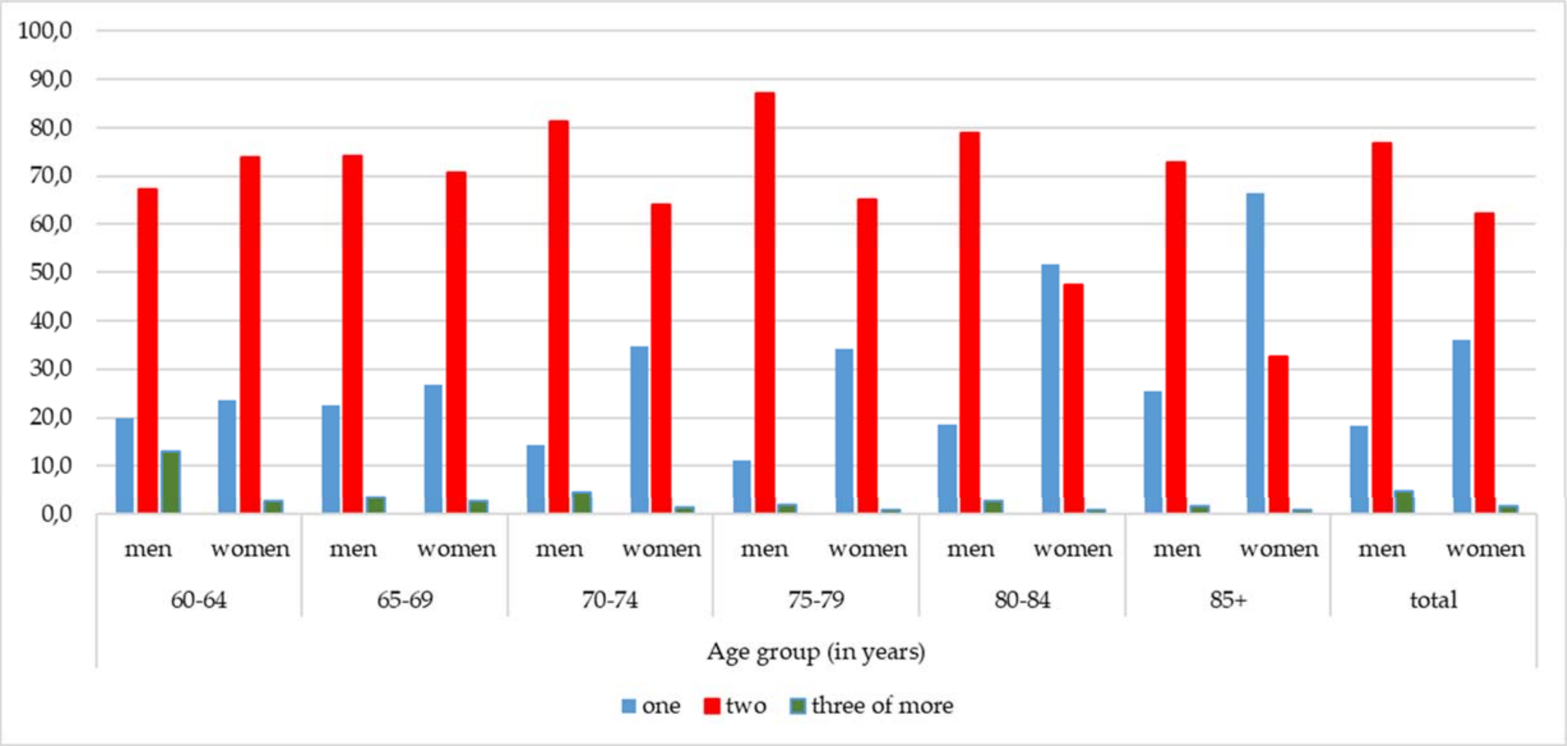

Supplement: Supplementary file 1 [file ijerph-19-11777-s001.zip › ijerph-1885865-supplementary.pdf]
